# Effects of fenofibrate on angiopoietin-like 3/4/8 proteins and apolipoprotein A5

**DOI:** 10.1016/j.jlr.2026.101050

**Published:** 2026-04-27

**Authors:** Ye Yang, Sydney Smith, Yan Q. Chen, Hongxia Li, Eugene Y. Zhen, John H. Sloan, Robert W. Siegel, Yuewei Qian, Yi Wen, Hyesoo Jung, Julia L. Scheithauer, Sander Kersten, Stephen G. Young, Robert J. Konrad

**Affiliations:** 1 Lilly Research Laboratories, Eli Lilly and Company, Indianapolis, IN, USA; 2 Department of Medicine, University of California, Los Angeles, Los Angeles, CA, USA; 3 Department of Human Genetics, University of California, Los Angeles, Los Angeles, CA, USA; 4 Nutrition, Metabolism, and Genomics Group, Division of Human Nutrition and Health, Wageningen University, The Netherlands; 5 Division of Nutritional Sciences, Cornell University, Ithaca, NY, USA

**Keywords:** APOA5, ANGPTL3/8, ANGPTL3, ANGPTL4/8, CD-ANGPTL4, fenofibrate

## Abstract

Fenofibrate and other fibrates are peroxisome proliferator–activated receptor alpha agonists that are used to lower plasma triglyceride (TG) levels. Although fibrates are effective in decreasing TG, their ability to reduce adverse cardiovascular events and cardiovascular mortality in clinical trials has been disappointing. Peroxisome proliferator–activated receptor alpha agonists influence the expression of dozens of genes, but the mechanisms by which they lower TG levels are incompletely understood. Apolipoprotein A5 (APOA5) and angiopoietin-like proteins 3, 4, and 8 (ANGPTL3/4/8) are important regulators of intravascular TG metabolism. To explore if their regulation might explain the TG-lowering effect of fibrates, we examined the impact of fenofibrate on the expression of APOA5 and the ANGPTL3/4/8 proteins in mice and humans. In WT mice, fenofibrate reduced plasma TG levels, increased *Angptl4* and *Angptl3* transcripts in the liver, and reduced *Angptl8* and *Apoa5* transcripts. Fenofibrate also decreased plasma APOA5 levels and increased levels of ANGPTL3, ANGPTL3/8, ANGPTL4/8, and the C-terminal domain of ANGPTL4 (CD-ANGPTL4). These changes would be predicted to increase rather than decrease TG levels. The TG reduction by fenofibrate was maintained in *Apoa5*-deficient mice, further indicating that APOA5 is not involved in TG lowering by fenofibrate. In humans, fenofibrate reduced TG without increasing APOA5 levels or reducing ANGPTL3/8 levels. In addition, fenofibrate treatment increased levels of ANGPTL3, ANGPTL4/8, and CD-ANGPTL4. The collective human and mouse data suggest that APOA5 and ANGPTL3/4/8 proteins do not mediate fenofibrate-induced TG lowering. Our findings are noteworthy because elevated levels of ANGPTL3, ANGPTL4/8, and CD-ANGPTL4 are associated with increased cardiovascular mortality.

The intravascular processing of triglyceride (TG)-rich lipoproteins (TRLs) by lipoprotein lipase (LPL) is responsible for the delivery of fatty acids to oxidative tissues (e.g., heart, brown adipose tissue, skeletal muscle) for fuel and to white adipose tissue for storage (1, 2). TRL processing reduces plasma TG levels, and the efficiency of TRL processing is controlled by multiple regulatory proteins. Apolipoprotein C2 binds to sequences surrounding LPL’s catalytic pocket, stabilizes the conformation of LPL’s catalytic domain, and increases LPL’s TG hydrolase activity (3, 4). A deficiency of apolipoprotein C2 causes severe hypertriglyceridemia (chylomicronemia) (5, 6).

Angiopoietin-like proteins 3, 4, and 8 (ANGPTL3/4/8) play critical roles in modulating LPL activity (7, 8, 9, 10, 11, 12, 13, 14, 15, 16, 17, 18, 19, 20, 21, 22). In particular, the atypical angiopoietin-like protein ANGPTL8, which has minimal ability to inhibit LPL on its own, differentially regulates the activities of the LPL inhibitors ANGPTL3 and ANGPTL4 in a tissue-specific and calorically sensitive manner (7, 8, 9, 13, 14, 15, 16, 17, 19, 20, 21, 22). After feeding, the liver secretes increased amounts of a complex composed of one molecule of ANGPTL8 and two molecules of ANGPTL3 (ANGPTL3/8) (23). This ANGPTL3/8 complex is a more potent LPL inhibitor than ANGPTL3 alone and acts in an endocrine manner to inhibit LPL in oxidative tissues (19, 20, 21, 22, 23). Mechanistically, ANGPTL3/8 promotes the unfolding of LPL’s catalytic domain in a manner like that of the potent LPL inhibitor ANGPTL4 (24), resulting in irreversible LPL inactivation, reduced amounts of LPL within capillaries, and reduced intravascular processing of TRLs (19, 20, 21, 22, 23, 24, 25).

Blocking the activity of the ANGPTL3/8 complex with an inhibitory antibody markedly reduces plasma TG levels (25, 26, 27). Apolipoprotein A5 (APOA5), a low-abundance plasma protein discovered in 2001 (28, 29, 30), reduces TG levels by binding to ANGPTL3/8 and suppressing ANGPTL3/8’s ability to unfold and inactivate LPL (25, 31). APOA5 also regulates TG levels by reducing circulating ANGPTL3/8 concentrations (25, 32, 33, 34). A deficiency of APOA5 causes severe hypertriglyceridemia (35, 36, 37, 38, 39, 40, 41, 42, 43).

After feeding, ANGPTL8 also forms a 1:1 complex with ANGPTL4 (ANGPTL4/8) that acts in an autocrine/paracrine manner in white adipose tissue to reduce the ability of ANGPTL4 to unfold and inhibit LPL (19, 20, 22). In white adipose tissue during the fed state, ANGPTL4/8 binds to LPL and protects it from ANGPTL4-mediated LPL unfolding, thereby preserving LPL’s catalytic activity (20). After feeding, ANGPTL4/8 binds tissue plasminogen activator and plasminogen to generate plasmin, which acts locally to cleave ANGPTL4/8 itself as well as ANGPTL3/8 so that LPL can be maximally active (44, 45). ANGPTL4/8 is also secreted by hepatocytes and is present in plasma at levels similar to those of ANGPTL3/8 (20), although the function of circulating ANGPTL4/8 has not been elucidated. Plasmin cleavage of ANGPTL4/8 in the adipose tissue generates a C-terminal domain-containing fragment of ANGPTL4 (CD-ANGPTL4) as does furin cleavage of ANGPTL4 in the liver and potentially other tissues as well (44, 45, 46). The relative contributions of different tissues to the circulating concentrations of ANGPTL4/8 and CD-ANGPTL4 are not currently known.

Understanding the regulation of plasma TG metabolism is important because the efficiency of intravascular TRL processing has been linked to the risk of cardiovascular disease (47, 48, 49). Loss-of-function mutations in *LPL* and *APOA5* increase plasma TG levels and increase the risk of coronary heart disease (50, 51), whereas loss-of-function mutations in *ANGPTL3*, *ANGPTL4*, and *ANGPTL8* reduce TG levels and lower the risk of cardiovascular events (46, 52, 53, 54, 55, 56, 57, 58). In addition to genetic observations, epidemiological studies have revealed that circulating levels of the ANGPTL3/4/8 proteins are linked to cardiovascular mortality. For example, elevated levels of the ANGPTL3/8 complex are associated with an atherogenic lipid profile (59). High levels of ANGPTL3, ANGPTL4/8, and CD-ANGPTL4 are associated with the progression of coronary artery atherosclerosis, coronary events, and increased cardiovascular mortality (60, 61).

Fibrates are effective in reducing plasma TG levels, but their ability to lower cardiovascular mortality has been disappointing (62, 63, 64, 65, 66, 67, 68). In a recent double-blind, randomized, placebo-controlled study of patients with type 2 diabetes, mild-to-moderate hypertriglyceridemia, and low HDL cholesterol levels, a potent fibrate (pemafibrate) reduced plasma TG levels by 26% but did not reduce the frequency of adverse cardiovascular events (69, 70, 71, 72). The reason for the failure to reduce cardiovascular events is not clear and has triggered considerable discussion (72).

Fibrates regulate TG metabolism by activating the transcription factor PPARα. While fibrates affect the expression of dozens of genes, the mechanism(s) by which they lower plasma TG levels is not fully understood (73). Fibrates increase the post-heparin levels of LPL in plasma (74) and are thought to alter the expression of LPL regulatory proteins (e.g., APOC3 and ANGPTL4) (75, 76, 77, 78, 79). PPARα agonists increase *ANGPTL4* expression in rat hepatoma FAO cells, human HepG2 cells, human primary hepatocytes, and “hepatocyte-humanized” mice (80, 81, 82). PPARα agonists have been reported to increase *APOA5* transcripts in HepG2 cells and cynomolgus macaque hepatocytes (83, 84); they have also been reported to increase *Apoa5* transcripts in mouse liver (85).

In the current studies, we investigated the impact of fenofibrate on the expression of ANGPTL3/4/8 proteins and APOA5 in mice and humans. In our studies, we analyzed transcript levels, and we also took advantage of immunoassays to measure circulating levels of ANGPTL3/4/8 proteins and APOA5. We had dual motivations for measuring the levels of APOA5 and the ANGPTL3/4/8 proteins during fenofibrate treatment. First, we hypothesized that the levels of APOA5 and ANGPTL3/8 could provide insights into fenofibrate’s ability to lower plasma TG levels. Second, we were interested in assessing the impact of fenofibrate on circulating levels of ANGPTL3, ANGPTL4/8, and CD-ANGPTL4 because epidemiological studies have uncovered a strong association between the levels of those proteins and cardiovascular mortality (60, 61).

## Materials and Methods

### Mice studies

All mouse studies were approved by UCLA’s Animal Research Committee. WT and *Apoa5*^−/−^ mice (FVB/NJ) have been described previously (25). WT mice (FVB/NJ) were purchased from The Jackson Laboratory. WT mice were fed a standard chow diet (LabDiet 5053, 20% protein, and 4.5% fat) ad libitum and were maintained in a facility on a 12-h light/12-h dark cycle. A fenofibrate diet (Inotiv TD.140506, 0.2% w/w) was prepared with fenofibrate (Sigma F6020) and the 2018 Teklad Global Rodent diet (18.6% protein and 6.2% fat). To assess the effects of fenofibrate in WT mice, two separate experiments were performed. In each case, male mice were used to avoid the potential confounding effect of sex on the expression of ANGPTL3/4/8 proteins and APOA5. In the first experiment, 10–12-week-old male mice were administered a 0.2% fenofibrate diet for 4 weeks. In the second experiment, 10–12-week-old male mice were fed a 0.2% fenofibrate diet or the standard chow diet for 4 weeks. Plasma samples were collected at baseline and at 2-weeks and 4-weeks time points. After 4 weeks of either the 0.2% fenofibrate diet or the chow diet, mice were perfused with PBS, and liver samples were collected, snap-frozen, and stored at −80°C. The effect of fenofibrate was also studied in 10-week-old male *Apoa5*^−/−^ mice. The *Apoa5*^−/−^ mice were fed the 0.2% fenofibrate diet for 4 weeks, and plasma samples were collected at baseline and endpoint. In all experiments, TG levels were measured with a serum TG kit (Sigma, TR0100).

### Quantitative RT-PCR

RNA from frozen liver biopsies was extracted with TRI reagent (Molecular Research); complementary DNA (cDNA) was prepared with random primers, oligo(dT), and SuperScript III (Thermo Fisher Scientific). Quantitative reverse transcription PCR was performed on triplicate samples with SYBR Green PCR Master Mix (Bioland) on a QuantStudio 5 Real-Time PCR System (Applied Biosystems). Transcript levels were measured with the comparative Ct method and normalized to transcript levels of a housekeeping gene (*Ppia*). We used the following forward and reverse primers:

*Apoa5:* CAGTTGGAGCAAAGGCGTGATG and CTCTCAAGGGTCCCAGCTTTTC.

*Angptl3:* ACGAAAAGGGCTTTGGGAGGCT and CGTAGTGCTTGCTGTCTTTCCAG.

*Angptl4*: CAAGACCATGACCTCCGTGG and CCGTGGGATAGAGTGGAAGTA.

*Angptl8*: GACTACAAGTGCAGCTGAGAGG and CAGTGAGAGCCCATAAGAGGTG.

### Human studies

Human plasma samples were obtained from a randomized, double-blind, placebo-controlled crossover intervention study designed to examine the effects of fenofibrate and fish oil treatment on inflammatory parameters, vascular function, and lipoprotein profiles in obese subjects (86, 87). The study was approved by the Medical Ethics Committee of Maastricht University and performed at Maastricht University. The study was registered under trial registration number EudraCT 2006-005743-28 and abided by the Declaration of Helsinki principles. In brief, 20 Caucasian subjects (10 men and 10 women with a BMI > 27 kg/m^2^) received 6 weeks of fish oil treatment, 6 weeks of fenofibrate treatment, and 6 weeks of placebo treatment (administered in random order with wash-out periods of >2 weeks between each treatment period).

Subjects given fish oil consumed fish oil (Marinol C-38, Lipid Nutrition) containing ∼3.7 g/d n-3 long chain polyunsaturated fatty acid (1.7 g/d EPA and 1.2 g/d DHA) and 200 mg cellulose (as a placebo for fenofibrate) per day. Subjects given fenofibrate consumed 200 mg fenofibrate (Lipanthyl, Fournier Laboratories) and eight capsules containing 80% high oleic sunflower oil (as a placebo for fish oil) per day. Subjects given placebo consumed eight high oleic sunflower oil capsules and 200 mg cellulose per day.

Doses of 200 mg/day and 3.7 g/day for fenofibrate and fish oil interventions, respectively, were selected because they have been shown to lower plasma TG levels. The power calculation was based on the primary outcome of the study, which was to detect a true difference of 0.20 mmol/L in plasma TG concentrations between the treatments with a power of 80%. Body weight in the subjects did not change between the treatment periods. Fenofibrate and fish oil decreased circulating TG levels by 27% and 13%, respectively (86, 87). Plasma samples were collected at baseline and after 5 and 6 weeks of each treatment for the analyses of ANGPTL3/4/8 proteins and APOA5.

Tissue biopsies were taken from 11 subjects for transcriptomic analyses. Muscle biopsies were taken from the *vastus lateralis*; subcutaneous adipose tissue was taken from the abdominal region, and peripheral blood mononuclear cells (PBMCs) were isolated from blood. These tissues were chosen because they were the most easily accessible in humans and because they might inform us about the effect of fenofibrate in the liver. While we would have liked to obtain liver samples from fenofibrate-treated subjects, a liver biopsy would have required the subjects to undergo a risky invasive procedure. RNA was isolated with Trizol and further purified using Qiagen RNeasy Micro columns (Qiagen). RNA was quantified with a Nanodrop ND 1000 spectrophotometer (Nanodrop Technologies), and RNA integrity was measured with an Agilent 2100 Bioanalyzer with RNA 6000 Nano chips (Agilent Technologies). RNA was labeled using a one-cycle cDNA labeling kit (MessageAmp II-Biotin Enhanced Kit; Ambion) and hybridized to Affymetrix human whole genome U133 plus 2.0 arrays. Hybridization, washing, and scanning of the Affymetrix arrays were performed according to Affymetrix protocols. Scans of the Affymetrix arrays were processed with packages from the Bioconductor project. Expression levels of genes were calculated using Gene Chip Robust Multi-Array Analyses.

### Immunoassays for APOA5, ANGPTL3, ANGPTL3/8, ANGPTL4/8, and CD-ANGPTL4

MesoScale Discovery (MSD) immunoassays for human APOA5, ANGPTL3, ANGPTL3/8, ANGPTL4/8, and CD-ANGPTL4 and for mouse APOA5 and ANGPTL3/8 have been described previously (20, 25, 33, 34, 60, 61, 88).

We created immunoassays for mouse ANGPTL3, ANGPTL4/8, and CD-ANGPTL4. For each assay, streptavidin-coated plates (MSD L15SA) were washed with 1× Tris-buffered saline containing 0.05% Tween-20 and blocked with 1× TBS containing 1% bovine serum albumin (BSA). After 1 h, wells were washed and coated with a biotinylated capture antibody in 1× Tris-buffered saline containing 0.05% Tween-20 with 0.1% BSA for 1 h. After washing, recombinant protein standards or mouse serum samples [diluted in assay buffer (50 mM Hepes pH 7.40, 150 mM NaCl, 1% Triton X-100, 5 mM EDTA, and 5 mM EGTA) supplemented with 1% BSA] were added to wells. After 2 h, wells were washed and incubated with a ruthenium-labeled detection antibody in a buffer containing 0.1% BSA for 1 h. After washing, 150 μl of 1× MSD read buffer (MSD R92 TC) were added to each well, and ruthenium electrochemiluminescence was quantitated with an MSD plate reader.

For the murine ANGPTL3 assay, a mouse ANGPTL3-specific polyclonal antibody (R&D Systems) was used for capture, and the same antibody was used for detection. For the murine ANGPTL4/8 assay, a mouse ANGPTL8-specific monoclonal antibody (mAb) was used for capture, and a mouse ANGPTL4-specific mAb was used for detection. For the murine CD-ANGPTL4 assay, a CD-ANGPTL4-specific mAb was used for capture, and a mouse CD-ANGPTL4-specific polyclonal antibody was used for detection.

### Immunoprecipitation and western blotting of murine CD-ANGPTL4

Equal amounts of plasma from fenofibrate-treated mice were pooled at 0, 2, and 4 weeks. Mouse CD-ANGPTL4 was immunoprecipitated from 50 μl of the pooled plasma (diluted 1:2 in PBS) using a mixture of mouse CD-ANGPTL4–specific monoclonal antibodies coupled to tosyl-activated beads (Thermo Fisher Scientific). Samples were incubated with beads overnight at 4°C. On the following day, the beads were washed with PBS, and bound proteins were eluted with 1% acetic acid, neutralized with 1 N NaOH, and boiled in lithium dodecyl sulfate sample buffer. Proteins were electrophoresed on a Novex 12% Bis-Tris gel. Recombinant mouse CD-ANGPTL4 protein (2.5 ng) was loaded in a separate lane as a standard. The separated proteins were transferred to a polyvinylidene fluoride membrane with an iBlot system (Thermo Fisher Scientific). The membrane was then incubated with a biotinylated mouse CD-ANGPTL4-specific polyclonal antibody, followed by an incubation with Alexa Fluor 680-conjugated streptavidin. Images were recorded with an Odyssey CLx imaging system (LI-COR Biosciences).

### Statistical analysis and data availability

Data are shown as the mean ± SD or mean ± SEM. For all murine and human immunoassays, MSD software (4.0.13) was used for curve fitting (5-parameter fit with 1/y^2^ weighting). Between-group differences were analyzed with a Student’s *t* test or with a one-way ANOVA followed by Tukey’s *post hoc* test or a mixed-effects model followed by Dunnett’s *post hoc* test. Statistical analyses were performed with Prism 10.6.0 (GraphPad). For all studies, *P* < 0.05 was considered significant. All data are available upon request.

## Results

### Fenofibrate treatment of mice reduces TG levels and lowers plasma APOA5 levels

**Fig. 1:**
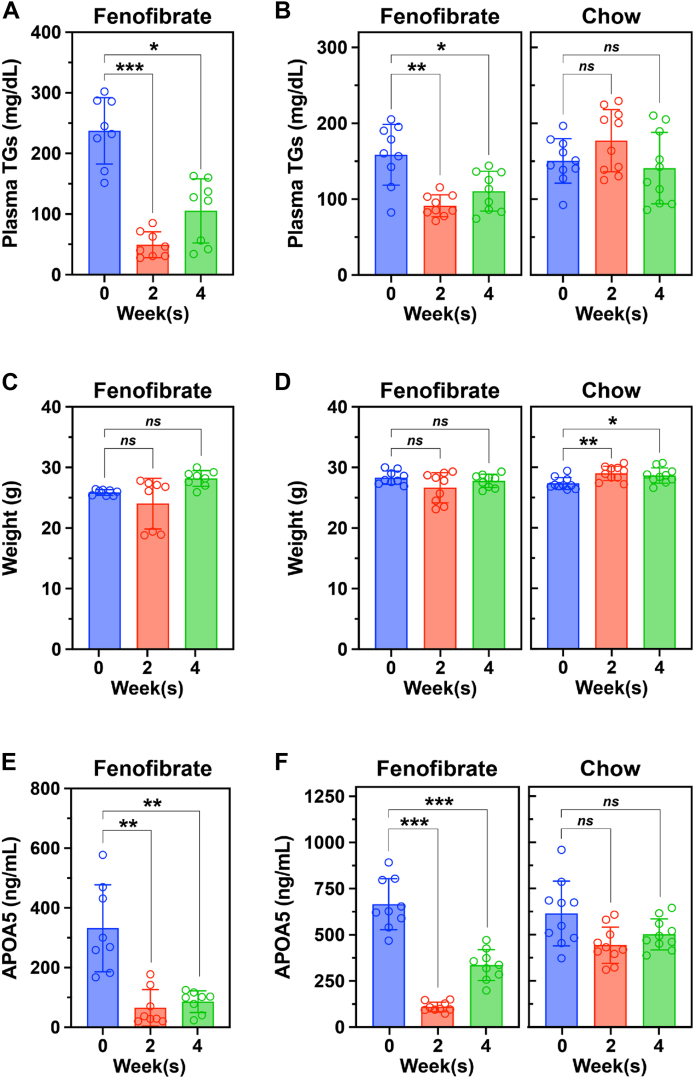
Fenofibrate decreases circulating TG and APOA5 concentrations in mice while not affecting their body weight. Male WT mice that were 10–12 weeks old (n = 8–10 mice/group) were administered a chow diet supplemented with 0.2% fenofibrate for 4 weeks (first experiment) or either a chow diet that was supplemented with 0.2% fenofibrate or a normal chow diet for 4 weeks (second experiment). Plasma TG levels in the first experiment (A) and second experiment (B), body weights in the first experiment (C) and second experiment (D), and APOA5 concentrations in the first experiment (E) and second experiments (F) were measured at baseline and after the mice had received 2 and 4 weeks of each diet. Each dot represents the plasma TG level, body weight, or circulating APOA5 concentration of each individual mouse at the given time point. Data are shown as the mean ± SD and were analyzed with a one-way ANOVA followed by Tukey’s HSD test (∗*P* < 0.05, ∗∗*P* < 0.01, ∗∗∗*P* < 0.001, *ns* not significant).

To examine the effect of fenofibrate in mice, we performed two experiments. In the first experiment (shown on the left side of Fig. 1), WT-mice were fed a chow diet supplemented with 0.2% fenofibrate for four weeks. In the second experiment (shown on the right side of Fig. 1), WT-mice were fed either a standard chow diet or a chow diet supplemented with 0.2% fenofibrate for four weeks. Plasma TG levels in fenofibrate-treated mice were 79% lower at week 2% and 56% lower at week 4; the chow diet had minimal effects on TG levels (Fig. 1A, B). Body weights in chow-fed mice increased modestly (by 5%–6%) over 4 weeks; weights in the fenofibrate-treated group did not change significantly (Fig. 1C, D).

APOA5 reduces plasma TG levels by suppressing ANGPTL3/8’s ability to inactivate LPL catalytic activity and its ability to reduce intracapillary levels of LPL (25, 31, 32, 33, 34). APOA5 also regulates the plasma levels of ANGPTL3/8; knocking out the *Apoa5* gene in mice or blocking APOA5 function with an inhibitory monoclonal antibody increases plasma levels of ANGPTL3/8 (34). PPARα agonists have been proposed to reduce plasma TG levels by increasing *APOA5* expression (83, 84, 85). In our studies, however, fenofibrate reduced APOA5 levels in the plasma by 84% at week 2 and by 74% at week 4; APOA5 levels did not change in mice fed the chow diet (Fig. 1E, F).

**Fig. 2:**
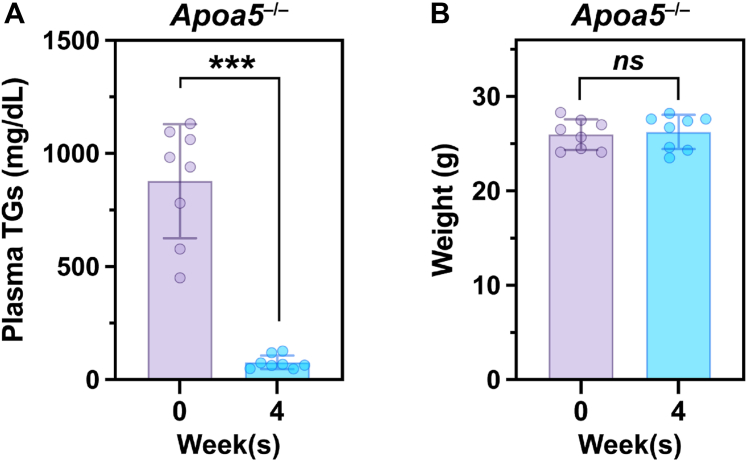
Fenofibrate decreases plasma TG levels in *Apoa5*^−/−^ mice. Ten-week-old male *Apoa5*^*−/−*^ mice (n = 8) were fed a 0.2% fenofibrate diet for 4 weeks. Plasma TG levels (A) and body weights (B) were determined at baseline and after 4 weeks. Each dot represents the plasma TG level or body weight in each mouse at the given time point. Data are shown as the mean ± SD and were analyzed with an unpaired, two-tailed Student’s *t* test (∗∗∗*P* < 0.001, *ns* not significant).

The fact that fenofibrate reduced circulating APOA5 levels suggested that the TG-lowering effects of fenofibrate were not mediated by APOA5. Consistent with that conclusion, fenofibrate markedly reduced plasma TG levels in *Apoa5*-deficient mice and did so without perturbing body weight (Fig. 2A, B).

### Impact of fenofibrate on plasma levels of the ANGPTL proteins

**Fig. 3:**
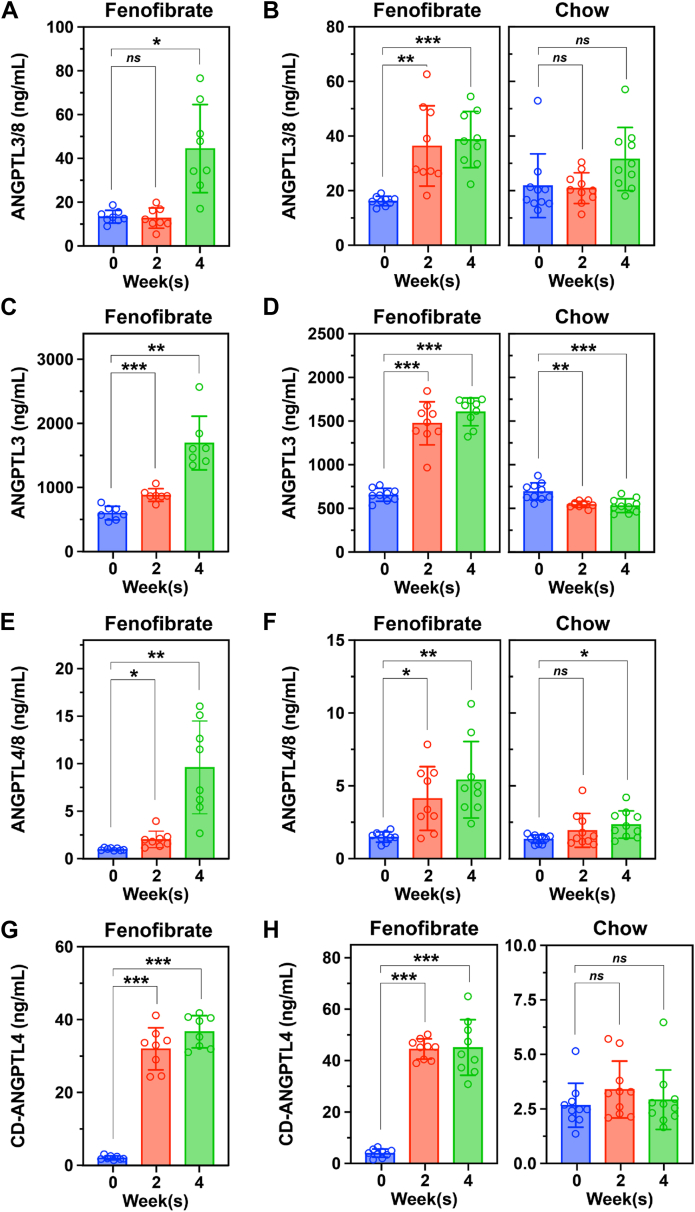
Fenofibrate increases circulating levels of ANGPTL3/8, ANGPTL3, ANGPTL4/8, and CD-ANGPTL4 in mice. Male WT mice that were 10–12 weeks old (n = 8–10 mice/group) were administered a chow diet supplemented with 0.2% fenofibrate for 4 weeks (first experiment) or either a chow diet that was supplemented with 0.2% fenofibrate or a normal chow diet for 4 weeks (second experiment). Plasma ANGPTL3/8 levels in the first experiment (A) and second experiment (B), ANGPTL3 levels in the first experiment (C) and second experiment (D), ANGPTL4/8 levels in the first experiment (E) and second experiment (F), and CD-ANGPTL4 levels in the first experiment (G) and second experiment (H) were measured at baseline and after the mice had received 2 and 4 weeks of each diet. The dots represent the plasma levels of each ANGPTL3/4/8 protein or complex measured in each individual mouse at the given time point. Data (mean ± SD) were analyzed with a one-way ANOVA followed by Tukey’s HSD test (∗*P* < 0.05, ∗∗*P* < 0.01, ∗∗∗*P* < 0.001, *ns* not significant).

In mouse models, we found that suppressing ANGPTL3/8 activity with an ANGPTL3/8-specific antibody increases intracapillary levels of LPL and sharply reduces plasma TG levels (25, 32, 33, 34). For that reason, we suspected that fenofibrate might lower plasma TG levels by reducing circulating levels of ANGPTL3/8. However, our two independent studies in which mice were fed a chow diet supplemented with 0.2% fenofibrate for four weeks showed that was not the case. Fenofibrate treatment of mice increased ANGPTL3/8 levels by 124% at week 2 and by 233% at week 4 while no significant effect was observed in the chow-fed control group (Fig. 3A, B).

In fenofibrate-treated mice, the levels of ANGPTL3 increased by 125% at week 2 and by 182% at week 4; in the chow-fed control group, ANGPTL3 levels decreased by 21% at week 2 and by 23% at week 4 (Fig. 3C, D). Fenofibrate increased ANGPTL4/8 levels by 178% at week 2 and by 868% at week 4; in the chow-fed control group, ANGPTL4/8 levels were unchanged at week 2 and were only 74% higher at week 4 (Fig. 3E, F). Fenofibrate increased CD-ANGPTL4 levels 14-fold at 2 weeks and 17-fold at 4 weeks; in the chow-fed control group, there were no significant changes in CD-ANGPTL4 levels (Fig. 3G, H).

**Fig. 4:**
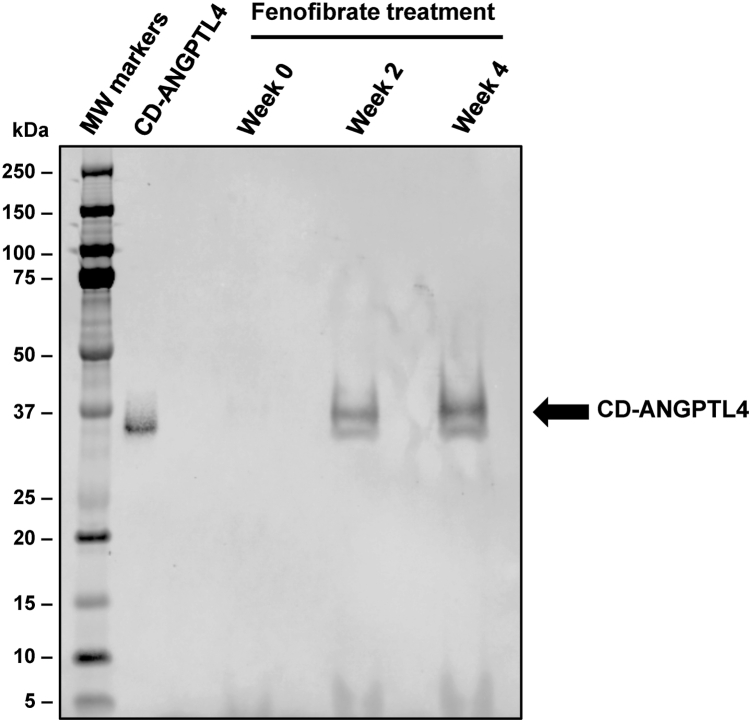
Assessing fenofibrate-induced increases in CD-ANGPTL4 levels by western blotting. Plasma from fenofibrate-treated mice at baseline (week 0), week 2, and week 4 were pooled (50 μl per sample) and immunoprecipitated with mouse CD-ANGPTL4-specific monoclonal antibodies. Proteins were separated by SDS-polyacrylamide gel electrophoresis, transferred to a PVDF membrane, and probed with a mouse CD-ANGPTL4-specific polyclonal antibody. PVDF, polyvinylidene fluoride.

To verify the fenofibrate-induced increases in CD-ANGPTL4 levels, plasma from fenofibrate-treated mice at baseline (week 0), week 2, and week 4 were pooled to generate a 50 μl sample at each time point. These samples were immunoprecipitated with a mixture of mouse CD-ANGPTL4-specific monoclonal antibodies, followed by western blotting with a mouse CD-ANGPTL4-specific polyclonal antibody. At baseline, an extremely faint band comigrated with the recombinant mouse CD-ANGPTL4 protein standard. At the 2-weeks and 4-weeks time points, the intensity of this band markedly increased (Fig. 4), confirming the CD-ANGPTL4 immunoassay findings (Fig. 3G, H).

### Fenofibrate increases Angptl3 and Angptl4 transcripts and reduces Angptl8 and Apoa5 transcripts in the liver

**Fig. 5:**
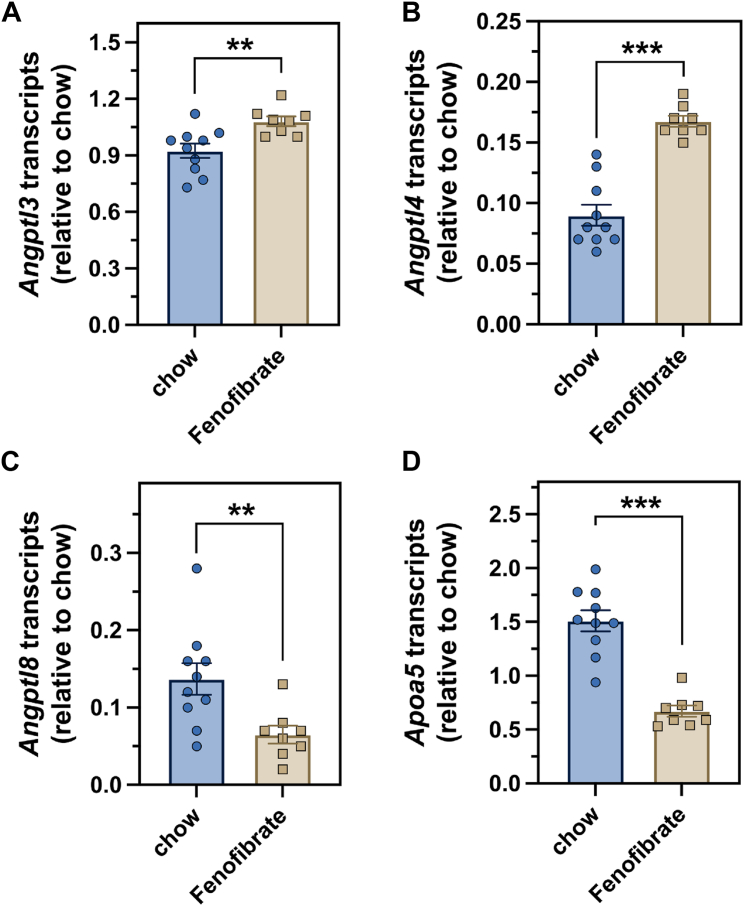
Fenofibrate increases murine hepatic *Angptl3* and *Angptl4* transcript levels while decreasing *Angptl8* and *Apoa5* transcript levels. Male WT mice that were 10–12 weeks old (n = 8–10 mice/group) were administered either a chow diet that was supplemented with 0.2% fenofibrate or a normal chow diet for 4 weeks. Following completion of the experiment, mice were perfused with PBS, and liver samples were collected and frozen for the subsequent measurement of transcript levels of *Angptl3* (A), *Angptl4* (B), *Angptl8* (C), and *Apoa5* (D). Each dot represents the transcript level in each individual mouse. Data (mean ± SEM) were analyzed with an unpaired, two-tailed Student’s *t* test (∗∗*P* < 0.01, ∗∗∗*P* < 0.001).

We assessed *Angptl3*, *Angptl4*, *Angptl8, and Apoa5* transcript levels in the livers of mice that were treated for four weeks with either a standard chow diet or a chow diet supplemented with 0.2% fenofibrate. After four weeks, *Angptl3* and *Angptl4* transcript levels were 17% and 82% higher, respectively, in fenofibrate-treated mice than in chow-fed mice, and *Angptl8* transcript levels were 53% lower in fenofibrate-treated mice than in chow-fed mice (Fig. 5A–C). *Apoa5* transcript levels in fenofibrate-treated mice were 56% lower than in chow-fed control mice (Fig. 5D).

### Fenofibrate increases ANGPTL3, ANGPTL4/8, and CD-ANGPTL4 levels in human subjects

**Fig. 6:**
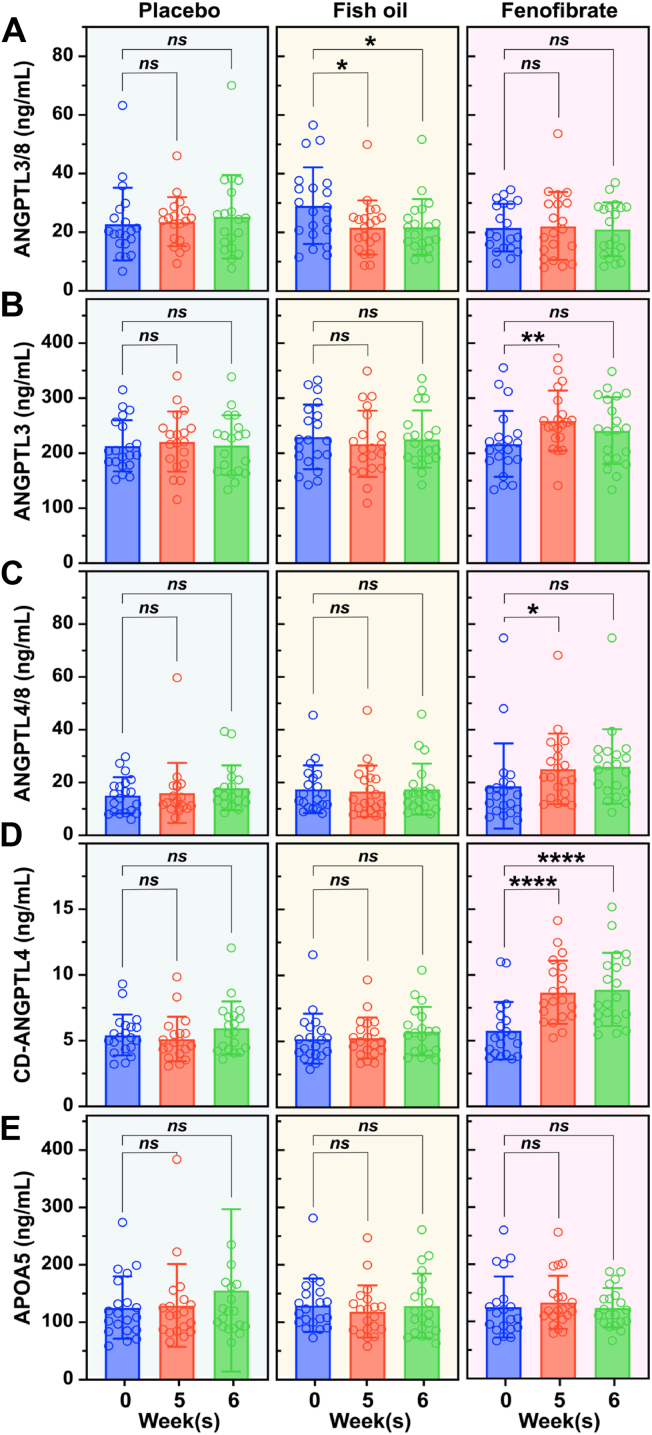
Fenofibrate increases circulating levels of ANGPTL3, ANGPTL4/8, and CD-ANGPTL4 in human subjects. Twenty human subjects were enrolled in a randomized, double-blind, placebo-controlled, crossover design in which they received 6 weeks of placebo treatment, fish oil treatment, or fenofibrate treatment with wash-out periods of at least 2 weeks between each 6-weeks treatment arm. Samples were obtained at baseline and after 5 and 6 weeks of each treatment, and levels of ANGPTL3/8 (A) ANGPTL3 (B), ANGPTL4/8 (C), CD-ANGPTL4 (D), and APOA5 (E) were measured. The dots represent the circulating levels of each ANGPTL3/4/8 protein or complex or APOA5 in each sample collected at the given time point. Data (mean ± SD) were analyzed using a mixed-effects model with a post-hoc Dunnett’s test (∗*P* < 0.05, ∗∗*P* < 0.01, ∗∗∗∗*P* < 0.0001, *ns* not significant).

**Fig. 7:**
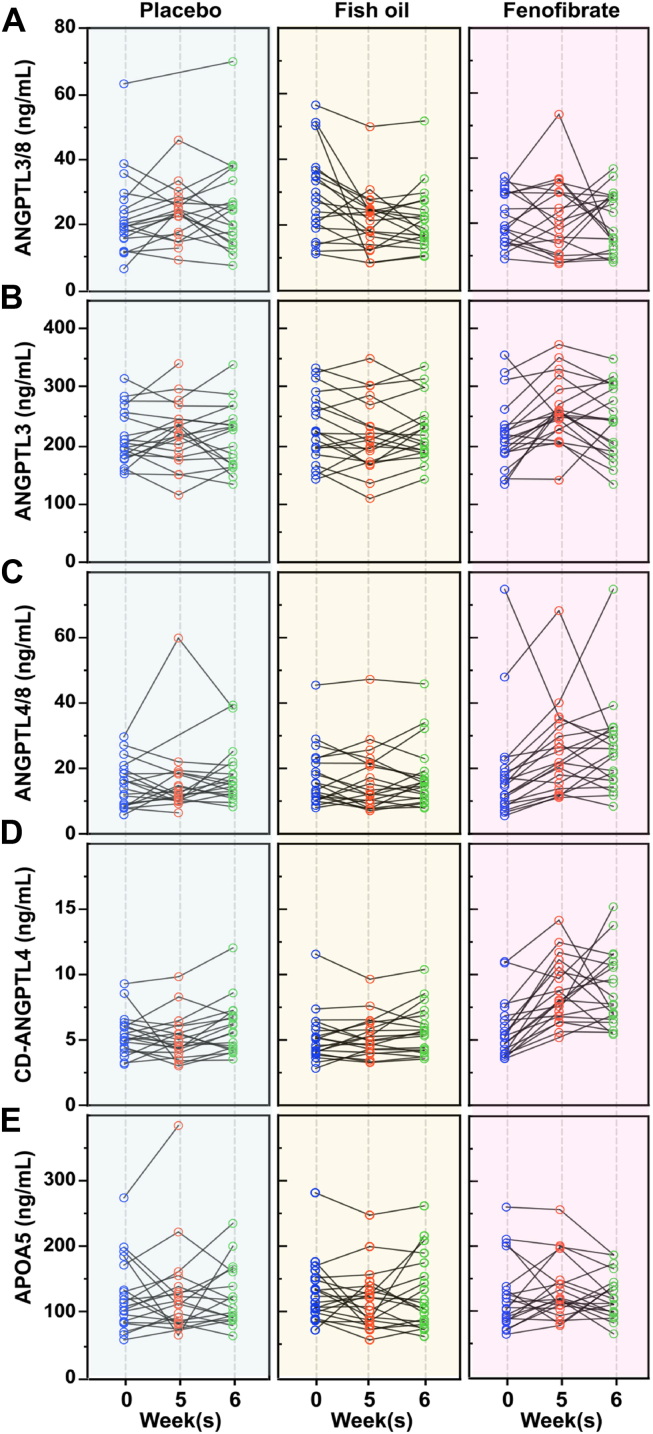
Spaghetti plots showing the effects of placebo, fish oil, and fenofibrate on APOA5 and ANGPTL3/4/8 proteins in individual human subjects. Spaghetti plots show the effects of placebo, fish oil, or fenofibrate on the circulating levels of ANGPTL3/8 (A) ANGPTL3 (B), ANGPTL4/8 (C), CD-ANGPTL4 (D), and APOA5 (E).

To determine the impact of fenofibrate administration on levels of the ANGPTL3/4/8 proteins and APOA5 in humans, we analyzed serum samples from 20 subjects enrolled in a randomized, double-blind, placebo-controlled crossover study in which each subject received 6 weeks of placebo, fish oil (1.7 g/d EPA and 1.2 g/d DHA), or fenofibrate (200 mg/d) (86, 87). Levels of ANGPTL3/8, ANGPTL3, ANGPTL4/8, CD-ANGPTL4, and APOA5 were measured at baseline and after 5 weeks and 6 weeks of each treatment. Placebo had no significant effects on any of the analytes; fish oil reduced ANGPTL3/8 levels by 25% after 5 and 6 weeks (Fig. 6A). Fenofibrate increased ANGPTL3 levels at 5 weeks and 6 weeks by 20% and 11%, respectively, but the increase at 6 weeks was not statistically significant (Fig. 6B). Fenofibrate also increased ANGPTL4/8 levels after 5 weeks and 6 weeks by 35% and 39%, respectively, but the increase at 6 weeks was not statistically significant (Fig. 6C). Fenofibrate increased CD-ANGPTL4 levels by 51% after 5 weeks (*P* < 0.0001) and 54% after 6 weeks (*P* < 0.001) (Fig. 6D). Neither fish oil nor fenofibrate altered circulating levels of APOA5 (Fig. 6E). Spaghetti plots for each analyte are shown in Figure 7.

### Fenofibrate treatment in human subjects did not affect ANGPTL4 transcript levels in adipose tissue, skeletal muscle, or PBMCs

**Fig. 8:**
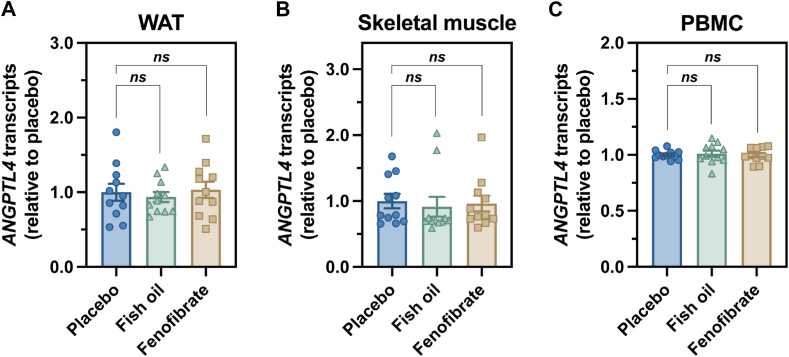
Fenofibrate does not increase *ANGPTL4* expression in white adipose tissue, skeletal muscle, or peripheral blood mononuclear cells (PBMCs) of human subjects. Expression levels of *ANGPTL4* mRNA were measured by transcriptome analysis in subcutaneous white adipose tissue (WAT) (A), *vastus lateralis* skeletal muscle (B), and PBMCs (C) collected from 11 human subjects after receiving 6 weeks of placebo, fish oil, or fenofibrate. Each dot represents the *ANGPTL4* expression level in each subject after the respective treatment period, and each line connects the *ANGPTL4* expression levels in the same subject. Data (mean ± SEM) were analyzed with a one-way ANOVA followed by Tukey’s HSD test (*ns* not significant).

We collected subcutaneous adipose tissue, *vastus lateralis* tissue, and PBMCs at the end of each 6-weeks treatment period. We found no consistent impact of fish oil or fenofibrate on *ANGPTL4* mRNA levels in those tissues (Fig. 8).

## Discussion

Fenofibrate promotes the intravascular lipolytic processing of TRLs and lowers plasma TG levels in both mice and humans, but the mechanisms are not fully understood (89). This study provides three key insights, although none of these findings can explain fenofibrate-induced decreases in plasma TG levels. First, we found no support for our earlier proposal (90) that fibrates might lower plasma TG levels by increasing APOA5 expression. We did not find higher plasma APOA5 levels during fenofibrate treatment in either mice or humans. In addition, fenofibrate sharply reduced TG levels in *Apoa5*^−/−^ mice. Second, we found no evidence that fenofibrate lowers TG levels by modulating ANGPTL3/8 levels. ANGPTL3/8 is a potent inhibitor of LPL, and blocking ANGPTL3/8 activity with an inhibitory antibody reduces plasma TG levels in both mice and humans (25, 26, 27). We found no evidence that fenofibrate reduces circulating ANGPTL3/8 levels in mice or humans. The third insight is that fenofibrate increases circulating levels of ANGPTL3, ANGPTL4/8, and CD-ANGPTL4 in mice and humans. That observation is noteworthy because elevated levels of those proteins are associated with increased cardiovascular mortality (60, 61).

APOA5 and ANGPTL3/8 regulate the amount of LPL along the luminal surfaces of capillaries (25, 31, 32, 33, 34). Because fenofibrate lowers plasma TG levels without increasing APOA5 levels or decreasing ANGPTL3/8 levels, we suspect that it probably works by increasing intracapillary LPL levels by an alternative mechanism. Supporting this idea, fibrates have been reported to increase amounts of LPL in post-heparin plasma (74) and reduce expression of APOC3 (an inhibitor of LPL activity) (75, 91, 92, 93). A limitation of our study is that post-heparin LPL activity levels and tissue-specific LPL activity levels were not measured. Further studies to examine the effects of fenofibrate on these parameters are encouraged. We suspect that fenofibrate lowers TG levels by increasing amounts of *LPL protein* along the luminal surfaces of capillaries. In future studies, this proposal could be tested with immunochemical methods (25, 32, 33, 34).

Earlier studies suggested that the TG-lowering effects of fish oil are distinct from those of fibrates (94, 95). Interestingly, our studies revealed that fish oil reduces ANGPTL3/8 levels; thus, it might lower plasma TG levels by reducing ANGPTL3/8 levels and thereby increasing intracapillary LPL levels.

In the current study, we found that fenofibrate increased *Angptl4* transcript levels in the liver and increased plasma levels of ANGPTL4/8 and CD-ANGPTL4 in mice. Fenofibrate also increased circulating ANGPTL4/8 and CD-ANGPTL4 levels in humans. The higher levels of ANGPTL4/8 and CD-ANGPTL4 in humans were not entirely surprising because pemafibrate had recently been reported to increase circulating ANGPTL4 levels (96). At this point, the tissue source of ANGPTL4/8 and CD-ANGPTL4 is not clear. ANGPTL4/8 could originate from hepatocytes or adipocytes; CD-ANGPTL4 could result from plasmin cleavage of ANGPTL4/8 or furin cleavage of ANGPTL4 (44, 45, 46).

Earlier studies of mice found that a PPARα agonist increased *Angptl4* expression in the small intestine and heart (97, 98). In humans, we suspect that the liver could also be relevant to the formation of some CD-ANGPTL4 because a PPARα agonist was reported to increase *ANGPTL4* expression in human hepatocytes by 2.7-fold (77, 78). Also, fenofibrate increased *ANGPTL4* transcripts by 2-fold in “humanized mice” in which mouse hepatocytes were ablated and replaced with human liver cells (82). We did not assess hepatic expression of *ANGPTL4* in our human subjects; however, we found no significant changes in *ANGPTL4* expression in subcutaneous adipose tissue, skeletal muscle, or PBMCs during fenofibrate treatment.

In mice, fenofibrate increased hepatic *Angptl4* transcripts by 82% but increased CD-ANGPTL4 levels by ∼15-fold. We do not understand this discrepancy. One possibility is that clearance of CD-ANGPTL4 may be reduced by fenofibrate. Another possibility is that extrahepatic ANGPTL4 production contributes to circulating CD-ANGPTL4. *Angptl4* expression is markedly induced by fenofibrate in the small intestine, and it is the most highly induced gene in the heart following PPARα activation with the PPARα agonist WY14643 (97, 98). *Angptl4* is also induced by fenofibrate in adipose tissue and skeletal muscle (99). Thus, it is difficult to attribute fenofibrate-induced increases in CD-ANGPTL4 to a specific tissue. Ultimately, we suspect that defining the source (or sources) of CD-ANGPTL4 will require studies with tissue-specific *Angptl4* KO mice.

In our studies, fenofibrate treatment of mice triggered high circulating levels of ANGPTL3 and ANGPTL4/8 in addition to the increased CD-ANGPTL4 levels. In humans, fenofibrate also increased plasma levels of ANGPTL3, ANGPTL4/8, and CD-ANGPTL4, although the magnitude of the increases was smaller than in mice. A potential explanation for the greater fenofibrate-responsiveness in mice could be the higher dose of fenofibrate given to mice. However, in “hepatocyte-humanized mice” (where livers contain both human and mouse hepatocytes), fenofibrate triggered a larger increase in mouse *Angptl4* transcripts than human *ANGPTL4* transcripts (82), suggesting that fenofibrate is more effective in inducing mouse *Angptl4* than human *ANGPTL4*. It is unlikely the differences are due to PPARα binding since the affinities of fenofibrate for mouse and human PPARα are similar (100). Inherent differences in mouse and human PPARα proteins might be important, as human PPARα-expressing mice do not exhibit hepatomegaly following PPARα activation (101). Further studies will be needed to explore these possibilities.

Large epidemiologic studies in humans have shown that elevated levels of ANGPTL3, ANGPTL4/8, and CD-ANGPTL4 are associated with the progression of coronary atherosclerosis, cardiovascular events, and cardiovascular mortality (60, 61). The increased cardiovascular risk was striking in subjects with elevated circulating levels of CD-ANGPTL4. At this point, we do not understand why CD-ANGPTL4 levels are associated with cardiovascular risk. CD-ANGPT4 could simply be a biomarker for a variety of metabolic abnormalities. Alternatively, it is possible that CD-ANGPTL4 acts directly (or indirectly) to promote atherogenesis or to reduce the stability of atherosclerotic plaques.

Because the circulating levels of ANGPTL3, ANGPTL4/8, and CD-ANGPTL4 are associated with an increased risk of cardiovascular disease (60, 61), the observation that fenofibrate increases the levels of these proteins is noteworthy and potentially concerning. It is conceivable that the higher levels of ANGPTL3, ANGPTL4/8, and CD-ANGPTL4 during fibrate therapy may offset the beneficial effects of lipid lowering and contribute to the disappointing results of fibrate therapy on cardiovascular mortality in human clinical trials. Additional prospective studies will be required to address that possibility.

### Data availability

All data supporting the conclusions of this article are contained within the article and will be made available to other researchers upon reasonable request to the corresponding authors.

### Declaration of generative AI and AI-assisted technologies in the writing process

No AI or AI-assisted technologies were used during the conduct of this study or in the writing and editing of the manuscript.

## Conflict of interest

All Lilly authors are full-time employees and shareholders of Eli Lilly and Company. The other authors declare no conflicts of interest.
